# Supplementary material for: A novel central nervous system-penetrating protease inhibitor overcomes human immunodeficiency virus 1 resistance with unprecedented aM to pM potency
Source: eLife. 2017 Oct 17;6:e28020. doi: 10.7554/eLife.28020 (PMC5644950; doi:10.7554/eLife.28020)
Supplement: Supplementary file 1. [file elife-28020-supp1.docx]

**Supplementary File 1.** Antiviral activity of five PIs and four NRTIs against laboratory-selected PI-resistant HIV-1 variants.

|  |  |  |  |  |  |  |  |  |  |
| --- | --- | --- | --- | --- | --- | --- | --- | --- | --- |
|  | Mean IC_50_ in nM ± SD (fold-change) | | | | | | | | |
|  | SQV | APV | IDV | NFV | TPV | AZT | 3TC | TDF | ABC |
| HIV_NL4-3_ | 12 ± 3 | 26 ± 8 | 18 ± 5 | 23 ± 5 | 330 ± 13 | 32 ± 6 | 280 ± 63 | 680 ± 260 | 670 ± 410 |
| HIV_SQV-5μM_ | >1,000 (>83) | 360 ± 18 (14) | >1,000 (56) | >1,000 (45) | 402 ± 22 (1) | ND^*^ | ND | ND | ND |
| HIV_APV-5μM_ | 65 ± 3 (5) | >1,000 (>38) | 415 ± 10 (23) | 413 ± 14 (18) | 310 ± 12 (1) | ND | ND | ND | ND |
| HIV_IDV-5μM_ | 440 ± 30 (37) | 300 ± 15 (12) | >1,000 (56) | >1,000 (45) | 386 ± 64 (1) | ND | ND | ND | ND |
| HIV_NFV-5μM_ | 40 ± 3 (3) | 71 ± 10 (3) | 450 ± 21 (25) | >1,000 (45) | 68 ± 63 (0.2) | ND | ND | ND | ND |
| HIV_TPV-15μM_ | >1,000 (>83) | >1,000 (>38) | >1,000 (56) | >1,000 (45) | >10,000 (30) | ND | ND | ND | ND |
| HIV_DRV_^R^_P20_ | 280 ± 140 (23) | >1,000 (>38) | >1,000 (56) | >1,000 (45) | 263 ± 74 (0.8) | >1,000 (>31) | >10,000 (>36) | 5700 ± 100 (8) | >10,000 (>15) |
| HIV_DRV_^R^_P30_ | 200 ± 11 (17) | >1,000 (>38) | >1,000 (56) | >1,000 (45) | 3,100 ± 200 (9) | >1,000 (>31) | >10,000 (>36) | >10,000 (>15) | >10,000 (>15) |
| HIV_DRV_^R^_P51_ | 270 ± 15 (23) | >1,000 (>38) | >1,000 (56) | >1,000 (45) | 3,300 ± 100 (10) | >1,000 (>31) | >10,000 (>36) | >10,000 (>15) | >10,000 (>15) |

*ND; not determined

The amino acid substitutions identified in reverse transcriptase of HIV_DRV_^R^_P51_ compared to the wild-type HIV_NL4-3_ include M41L, E44D, D67N, T69D, M184V, L210W, T215Y/F, D218E, and K219Q/N.

Numbers in parentheses represent fold changes in IC_50_s for each isolate compared to the IC_50_s for HIV_NL4-3_. All assays were conducted in triplicate, and the data shown represent mean values (± 1 standard deviation) derived from the results of three

independent experiments.
