# Supplementary material for: A novel central nervous system-penetrating protease inhibitor overcomes human immunodeficiency virus 1 resistance with unprecedented aM to pM potency
Source: eLife. 2017 Oct 17;6:e28020. doi: 10.7554/eLife.28020 (PMC5644950; doi:10.7554/eLife.28020)
Supplement: Supplementary file 2. [file elife-28020-supp2.docx]

**Supplementary File 2. Representative dose-response profiles of DRV, GRL-121, and GRL-142 against cHIV_NL4-3_^WT^, cHIV_NL4-3_^V32I^, cHIV_NL4-3_^G48V^, cHIV_NL4-3_^I50V^, and cHIV_NL4-3_^V82T^ are shown.** Three different attempts are shown in black, red, and blue.
