# Supplementary material for: A novel central nervous system-penetrating protease inhibitor overcomes human immunodeficiency virus 1 resistance with unprecedented aM to pM potency
Source: eLife. 2017 Oct 17;6:e28020. doi: 10.7554/eLife.28020 (PMC5644950; doi:10.7554/eLife.28020)
Supplement: Supplementary file 3. [file elife-28020-supp3.docx]

**Supplementary File 3. List of 37 HIVs used in antiviral assay and**

**amino acid sequences of the protease-encoding region of HIVs used in this study.**

|  |  |  |
| --- | --- | --- |
| Virus species |  |  |
| Wild-type HIV-1 clone | cHIV_NL4-3_^WT^ | Table 1- 4, 6 and Supplementary File 1 |
| Wild-type HIV-2 | HIV-2_ROD_ | Table 2 |
|  | HIV-2_EHO_ | Table 2 |
| *In vitro* PI-selected HIV-1 variants | HIV_SQV-5μM_ | Table 3 and Supplementary File 1 |
|  | HIV_APV-5μM_ | Table 3 and Supplementary File 1 |
|  | HIV_LPV-5μM_ | Table 3 |
|  | HIV_IDV-5μM_ | Table 3 and Supplementary File 1 |
|  | HIV_NFV-5μM_ | Table 3 and Supplementary File 1 |
|  | HIV_ATV-5μM_ | Table 3 |
|  | HIV_TPV-15μM_ | Table 3 and Supplementary File 1 |
|  | HIV_DRV_^R^_P20_ | Table 1, 3 and Supplementary File 1 |
|  | HIV_DRV_^R^_P30_ | Table 1, 3 and Supplementary File 1 |
|  | HIV_DRV_^R^_P51_ | Table 1, 3 and Supplementary File 1 |
| Recombinant clinical HIV-1 variants | _rCL_HIV_F16_ | Table 3 |
|  | _rCL_HIV_F39_ | Table 3 |
|  | _rCL_HIV_V42_ | Table 3 |
|  | _rCL_HIV_T44_ | Table 3 |
|  | _rCL_HIV_M45_ | Table 3 |
|  | _rCL_HIV_T48_ | Table 3 |
| HIV-1 clones carrying single amino acid substitution in PR region | cHIV_NL4-3_^L10F^ | Table 4 |
|  | cHIV_NL4-3_^L24I^ | Table 4 |
|  | cHIV_NL4-3_^D30N^ | Table 4 |
|  | cHIV_NL4-3_^V32I^ | Table 4 |
|  | cHIV_NL4-3_^L33F^ | Table 4 |
|  | cHIV_NL4-3_^M46I^ | Table 4 |
|  | cHIV_NL4-3_^I47V^ | Table 4 |
|  | cHIV_NL4-3_^G48V^ | Table 4 |
|  | cHIV_NL4-3_^I50V^ | Table 4 |
|  | cHIV_NL4-3_^I54M^ | Table 4 |
|  | cHIV_NL4-3_^I54L^ | Table 4 |
|  | cHIV_NL4-3_^I54V^ | Table 4 |
|  | cHIV_NL4-3_^L63P^ | Table 4 |
|  | cHIV_NL4-3_^V82A^ | Table 4 |
|  | cHIV_NL4-3_^V82I^ | Table 4 |
|  | cHIV_NL4-3_^V82T^ | Table 4 |
|  | cHIV_NL4-3_^I84V^ | Table 4 |
|  | cHIV_NL4-3_^L90M^ | Table 4 |

**
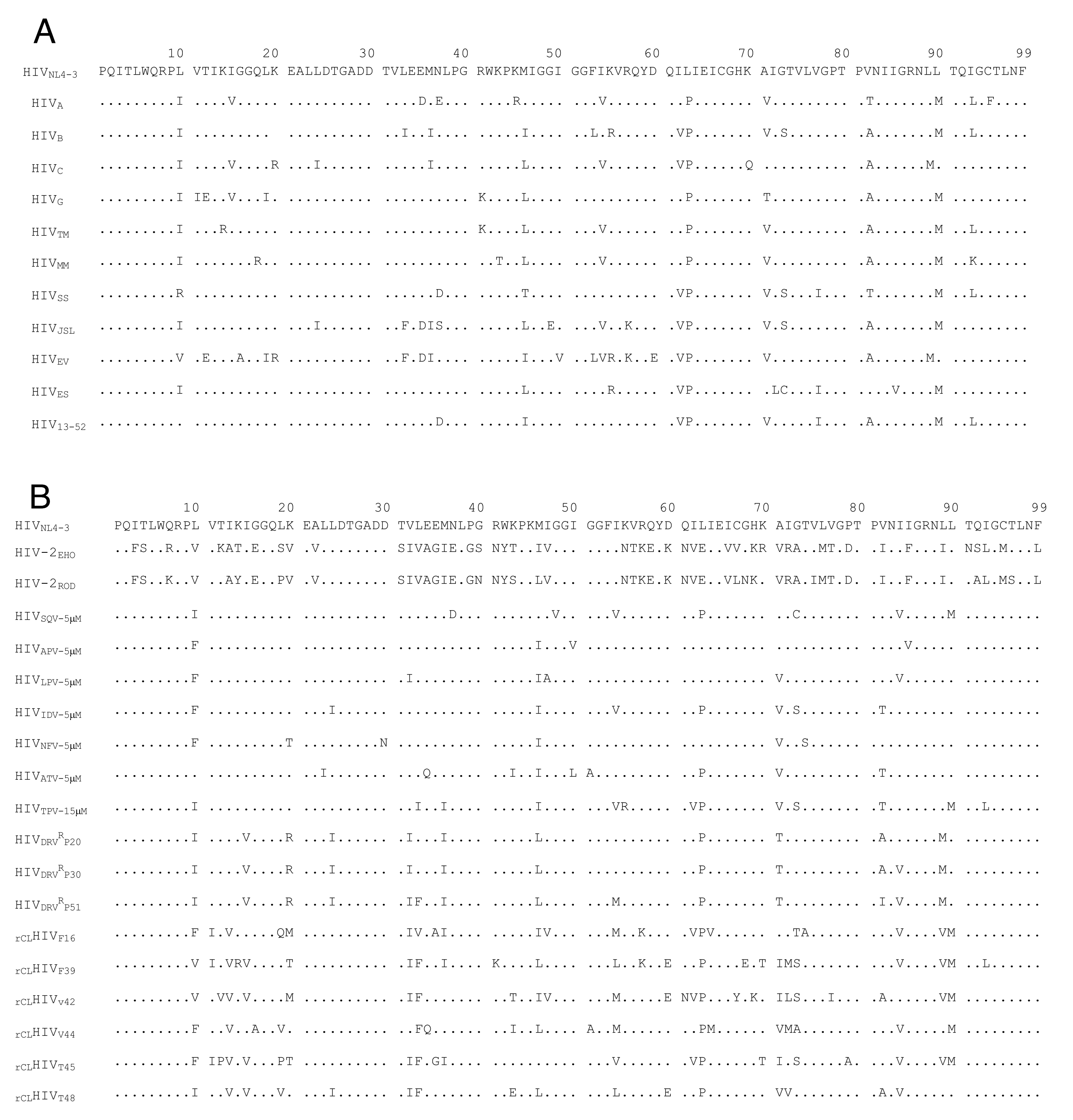
**

The consensus sequence of HIV_NL4-3_ is illustrated at the top as a reference. Identity with sequence at individual amino acid positions is indicated by dots. **(A)** The amino acid sequence of the eleven HIVMDRs. **(B)** The amino acid sequence of the PI-resistant HIV-1 variants used in drug susceptibility assay.
